# Anti-HER2 scFv-CCL19-IL7 recombinant protein inhibited gastric tumor growth in vivo

**DOI:** 10.1038/s41598-022-14336-1

**Published:** 2022-06-21

**Authors:** Haiqiang Zhang, Xueshuai Ye, Junye Wen, Ziqi Cai, Yang Li, Mengya Zhang, Li Shen, Jianhui Cai

**Affiliations:** 1 Department of Surgery, Hebei Medical University, 361 East Zhongshan Road, Shijiazhuang, 050017 Hebei China; 2 Department of Oncology &Surgery, Hebei General Hospital, 348 West Heping Road, Shijiazhuang, 050051 Hebei China; 3 Department of Surgery, Second Hospital of Hebei Medical University, Shijiazhuang, 050000 Hebei China; 4 Hebei Cell Therapy Technology Innovation Center, HOFOY Medicine Hebei Co., LTD, 238 Yangzi River Avenue, Shijiazhuang, 050000 China

**Keywords:** Molecular engineering, Cancer immunotherapy, Drug development, Targeted therapies

## Abstract

HER-2 targeted therapies, such as monoclonal antibodies (mAbs) and CAR-T cell therapy have been applied in the treatment of various of cancers. However, the anti-HER2 CAR-T cell therapy are limited by its expensive production procedure and fatal side effects such as cytokine storm or “On target, off tumor”. The application of anti-HER2 mAbs to the soild tumor are also plagued by the patients resistant with different mechanisms. Thus, the recombinant protein technology can be presented as an attractive methods in advantage its less toxic and lower cost. In this study, we produced a HER-2-targeting recombinant protein, which is the fusion of the anti-HER-2 single chain fragment variable domain, CCL19 and IL7 (HCI fusion protein). Our results showed that the recombinant protein can induce the specific lysis effects of immune cells on HER-2-positive gastric tumor cells and can suppress gastric tumor growth in a xenograft model by chemotactic autoimmune cell infiltration into tumor tissues and activated T cells. Taken together, our results revealed that the HCI fusion protein can be applied as a subsequent clinical drug in treating HER-2 positive gastric tumors.

## Introduction

Gastric carcinoma originates from mucosal epithelial cells located in the superficial layer of the gastricwall^1^.Due to its insidious onset, the diagnosis of gastric carcinoma is usually delayed, and the majority of gastric cancer patients are already in the advanced stage when diagnosed, among which 30%-40% of stage IV cases, are characterized by a poor clinical outcome and high mortality^2^.In recent years, tumor association antigen (TAA)-targeting therapies such as monoclonal antibodies and CAR-T cell therapy have been applied to treat gastric carcinoma^3–5^.

The proto-oncogene HER2, also known as ErbB2, plays an important role in the pathogenesis and clinical development of gastric and other tumor types^6,7^. Current studies have shown that HER2 over-expression exists in breast cancer, ovarian cancer, gastric cancer, prostate cancer and other tumors to varying degrees, and the size, stage, lymph node metastasis and prognosis of tumors are closely related to their expression intensity^8^.

Therefore, HER2 could serve as an ideal target for antitumor therapy by using CAR-T cells, and a series of preclinical studies applied HER2-specific CAR-T cells to treat gastric cancer^9–11^.However, CAR-T cells may lead “on target, off tumor” by targeting HER-2-positive normal tissues and cause fatal toxicity^12^.

The monoclonal antibody trastuzumab has been used to treat HER-2-overexpressing metastatic gastric cancer. Bang et al.^13^performed a Trastuzumab for Gastric Cancer (ToGA) phase III clinical trial by recruiting 584 patients with either inoperable locally advanced, recurrent, or metastatic cancers. The patients were treated with trastuzumab in combination with a fluoropyrimidine and cisplatin or chemotherapy alone. The patients treated with trastuzumab showed improvement of median overall survival (mOS,13.8 months vs. 11.1 months of chemotherapy alone) and overall response rate (ORR, 47% vs. 35% of chemotherapy alone), indicating that anti-HER2 therapy is a promising methods in treating gastric cancer. However, a consistent number of tumor patients become resistant to trastuzumab and several mechanisms have been described for that phenomenon. On the one hand, HER family members such as EGFR and MET can compensate for HER2 blockade. The gastric cancer cells can upregulation the EGFR and MET to activate SRC to activates PI3K signaling when received anti-HER2 therapy^14^. On the other hand, the anti-HER2 therapy can induce the loss of HER2 expression due to the HER2 signaling inhibitory and ADCs effects^15^, suggesting that novel HER-2 targeting therapy is urgently.

As CCL19 is a chemoattractant for T cells and DCs, and IL-7 is known to enhance the proliferation and survival of T cells^16^, CAR-T cells that produce IL-7 and CCL19 can recruit T cells and DCs to tumor tissues and enhance T cell viability in the tumor immune-inhibitory microenvironment (TME),which could improve the therapeutic effects of CAR-T cells against solid tumors^17^. However, the new generation of CAR-T cells may also lead to more serious side effects.

Thus, we wanted to generate a Recombinant protein that was in tandem with HER-2-specific single-chain variable fragment (scFv), CCL19 and IL-7(HCI fusion protein).Our results demonstrated that the HCI fusion protein can be stably obtained from transfected HEK-293 T cell strains. In addition, it can specifically target the HER-2 antigen molecule and induce immune cell infiltration into tumor tissues with activated effector T cells at the same time. Here, we demonstrated that the HCI fusion protein is capable of inducing immune cells to eradicate HER-2-positive cells both in vitro and in vivo, which showed promising safety and efficacy in future clinical applications.

## Material and methods

All methods were carried out in accordance with relevant guidelines and regulations and all experimental protocols were approved by a Hebei Medical University.

### Animals

All animal experiments were conducted under the approval of Hebei Medical University Animal Care and Use Committee, Hebei, China. All animal experiments were conducted under the approval of the Hebei Medical University Animal Care and Use Committee, Hebei, China. All NOD/SCID (non-obese diabetic/severe combined immunodeficiency) mice used in this study were healthy males (6–8 weeks of age), which were randomly assigned to experimental or control groups.

### Cell lines

HEK-293 T and the human gastric cancer cell NCI-N87 and SGC7901 were kindly provided by the Scientific Research Center of Hebei Medical University. HEK-293 T cells were cultured in DMEM (Gibco, USA) supplemented with 10% FBS (fetal bovine serum, Gibco, USA), whereas NCI-N87 and SGC7901 cells were cultured in RPMI-1640 medium (Gibco, USA) supplemented with 10% FBS. All of the cells were maintained at 37℃ in 5% CO_2_.

### Construction of plvx-HIC vector

**Figure 1:**
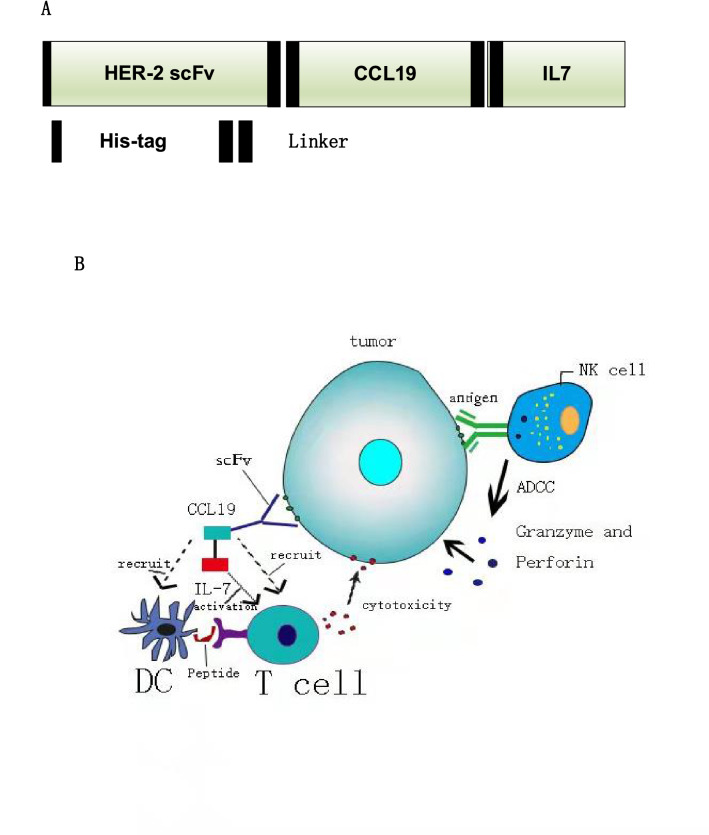
Schematic diagram of the HCI fusion protein killing effects. (**A**) Illustrative representation of the HCI fusion protein construct. (**B**) Schematic diagram of the HCI fusion protein killing effects when targeting HER-2 antigen positive tumor cells. Unlike the ADCC effects which mainly induced by NK cells, the HCI protein can recognized the HER-2 antigen(the function of HER-2 scFv) and recruit DC and T cells to infiltrated to the tumor site (the function of CCL19 part)and activated the effector cell (the function of IL-7 part and the DC cells which recruit by the HIC).

The amino acid sequence of an anti-HER-2 antibody’s single chain fragment variable (scFv) was as previously described^18^. The human IL-7 and human chemokine CCL19 amino acid sequences were obtained from GenBank. The HIC fusion protein expression construct was generated by fusing the anti-HER-2 scFv with CCL19 and IL-7, and each element was concatenated with the G34S linker^19^ (Fig. 1a). This whole construct containing the XhoI/HindIII restriction site was cloned into pcDNA3.1, subcloned into the pLVX-puro vector, which contains a GFP cassette, and then used for the production of lentivirus packaging.

### Lentivirus production

Transduction of the HIC-fusion protein-expressing lentivirus vectors into HEK-293 T packaging cells was conducted as previously described^20^. Briefly, lentivirus was produced by co-transfecting HEK-293 T cells with the packaging vector psPAX2, the envelope vector pCL-VSVG and each of the Lenti V2 vectors. Twenty-four hours after transfection, the culture medium was replaced with fresh DMEM supplemented with 10% FBS. The lentivirus in the supernatant was harvested 72 h after transfection and used for gene transduction.

### Expression and purifcation of HIC-fusion protein

Another resuscitated HEK-293 T cell line was plated in 6-well plants and infected with viral supernatants in the presence of polybrebe (GeneChem, China), followed by additional incubation for 2 days. Then, the DMEM growth medium was changed to selection medium containing 1 μg/ml puromycin for 7 days. Cells were then expanded and frozen once the cells grew normally in selection medium to obtain HEK-293 T cells stably expressing the HCI fusion protein (HCI-293 T).

Next, protein samples from the lysates of HCI-293 T cells were purified by an ion exchange chromatography column (HiTrap™ DEAE FF; loading buffer: Tris-HCl 0.02 mol/l, pH 8.8; and elution buffer: 50 mM/100 mM/200 mM/500 mM NaCl Tris-HCl 0.02 mol/l, pH 8.8; GE Healthcare Life Sciences, Chalfont, UK) according to the manufacturer’s instructions. Protein concentration was measured with a BCA assay kit (Solarbio, China).

### Western blot analysis of HIC-fusion protein

The total protein in HCI-293 T cellswas extracted by RIPA lysis buffer containing protease inhibitor. The protein concentration was determined by the BCA method (Thermo Fisher Scientific, USA) following the manufacturer’s instructions.

Proteins were separated on a 12% SDS-PAGE gel and transferred to polyvinylidene fluoride (PVDF) membranes. The PVDF membrane was blocked in 5% skim milk for 2 h and then incubated with the primary antibody at 4℃overnight. After washing the PVDF membrane, it was incubated with the corresponding secondary antibody for 2 h at room temperature. Proteins were visualized on a gel imager using a SuperSignalTM West Pico PLUS chemiluminescentsubstrate (Thermo Fisher Scientific,USA). The primary antibodies used were as follows: anti-His tag (1:1000, Abcam)and anti-β-actin (1:2000, Abcam).

### Preparation of human PBMCs cells

Peripheral blood samples were obtained from healthy donors at Hebei Blood Center, Hebei, China. Informed consent was obtained from all donors in accordance with the guidelines approved by Hebei Medical University, Hebei,China. PBMCs (peripheral blood mononuclear cells) were isolated from whole blood samples by Ficoll-Paque density gradient centrifugation (GE Healthcare, USA). PBMCs from the interphase were collected, washed with complete medium three times and then cultured in RPMI-1640 complete medium supplemented with 10% FBS at 37 ℃ with 5% CO_2_ overnight.

### Flow cytometry analysis

For flow cytometry analysis, BD FACS (Fluorescence-Activated Cell Sorting) Aria II (BD Bioscience) was used. HER-2 protein expressed on NCI-N87 and SGC7901cells was detected with FITC-labeled mouse anti-HER2 lgG1 antibody (Santa Cruz, USA); the isotype control group was stained with FITC-labeled lgG1 (Santa Cruz, USA).

The binding capability of the HCI fusion protein to HER-2-positive tumor cells was determined as follows: 1 × 10^6^ NCI-N87 or SGC7901 cells were harvested and placed in a 2 ml tube and washed 3 times with 3 ml of ice-cold PBS containing 4% BSA (bovine serum albumin). After washing, the cells were resuspended in 200 μl of ice-cold wash buffer and incubated with 1 μg of HIC fusion protein at 4 °C for 45 min. The cells were washed with 3 ml of ice-cold wash buffer 3 times and then incubated in the dark with 10 μl of PE-labeled MIP-3 beta monoclonal antibody (Thermo Fisher Scientific, USA) at 4℃ for 30 min. After washing an additional 2 times, the cells were examined for CCL19 on the cell surface by FACS. To evaluate the humanized immune system in NOD/SCID mice, the phenotype of CD45 positive memory associated PBMCs in the mouse veins was detected with human CD3-FITC, CD19-APC-A, CD56-PE-A, CD33-PE-A (all from BD Bioscience). All of the processes were performed according to the manufacturer’s instructions, and the FACS data were analyzed using FlowJo 8.1.1 software (FlowJo, LLC).

### Cytotoxicity assay

5 × 10^4^ NCI-N87 or SGC7901 cells in 100 µl RPMI complete medium/well were planted in flat-bottomed 96-well plates, respectively, Normal saline group (NS group) was added 10 μl normal saline, Trastuzumab group was added 10 μl 1 μg/μl Trastuzumab, HCI fusion protein group was added with 10 μl 1.5 μg/μl HCI fusion protein, then the PBMC cells were added at the increasing effector-to-target cell ratios (E:T = 5:1,10:1,20:1) and the target cells lysis were analyzed using Cell Counting Kit-8 (DOJINDO, Japan) according to the manufacturer’s instruction.

### ELISA

ELISA was performed with the same procedure as the cytotoxicity assay except that the E:T ratio was fixed at 20:1. After 24 h, we harvested supernatants and then measured the cytokine release levels of IL-2 and IFN-γ using ELISA kits (eBioscience,USA). For the in vivo experiment, we collected 100 μl peripheral blood from xenograft mice when the mice were sacrificed.

### Humanized immune system reconstruction in NOD/SCID mice, tumor challenge and treatment

To reconstruct the humanized immune system in NOD/SCID (non-obese diabetic/severe combined immunodeficiency) mice, mice were injected with 4 × 10^7^ PBMCs for 4 weeks as previously described^21^ and all methods are reported in accordance with arrive guidelines (https://arriveguidelines.org) for the animal experiments. Four weeks later, NOD/SCID mice with a humanized immune system were subcutaneously inoculated with 5 × 10^6^ NCI-N87 cells/0.5 ml PBS to develop tumor xenografts.

Approximately 7 days later, when the tumor volume reached 150 mm^3^, the mice were randomly divided into three groups and the three groups were treated with initial load dose: NS group: 0.5 ml/ mouse, Trastuzumab group: 4 mg/kg/0.5 ml/ mouse, HCI Fusion Protein group: 5 mg/kg/0.5 ml/mouse. The maintenance doses were set as follows: NS group: 0.5 ml/mouse, trastuzumab group: 2 mg/kg/0.5 ml/mouse, and HCI fusion protein group: 2.5 mg/kg/0.5 ml/mouse. The treatment was repeated once a week, the tumor size was measured using a Verniercaliper every other day, and the tumor volume was calculated as volume = (length × width^2^)/2. Mice were sacrificed 4 weeks after the initial treatment. ELISA (eBioscience, USA) was used to detect IFN-γ and IL-2 secretion in mouse blood serum.

### Histopathological and immunofluorescence assays

To examine the pathology and cell infiltration, hematoxylin and eosin (H&E) staining and immunofluorescence staining were performed. The paraffin sections were first dewaxed and stained with hematoxylin. Sections were then dehydrated in an ethanol gradient and stained with eosin. Finally, the sections were sealed and observed and imaged under an optical microscope (Tokyo, Japan,Nikon). For immunofluorescence staining, paraffin sections were dewaxed and placed in antigen retrieval solution (pH8.0). AutoFluo Quencherwas added to the sections and incubated with BSA for 30 min for serum blocking. The primary antibody was added dropwise to the sections and incubated at 4 °C overnight. The sections were washed with PBS (pH7.4) and then incubated with secondary antibody for 50 min. Sections were incubated with 4ʹ,6-diphenyl-2-phenylindole (DAPI) for 10 min to re-stain cell nuclei. Sections were observed, and images were acquired under a fluorescence microscope (Tokyo, Japan, Nikon). The primary antibodies used were as follows: anti-DEC205 (1:1000, Abcam) and anti-CD3 (1:200, Abcam).These experiments were independently repeated three times.

### Statistical analysis

All results are expressed as the mean ± SEM (standard error of the mean). Statistical significance was assessed using ANOVA (one-way analysis of variance). A value of *P* < 0.05 was considered statistically significant.

## Results

### The generation of HCI fusion protein from HCI-293 T cells

**Figure 2:**
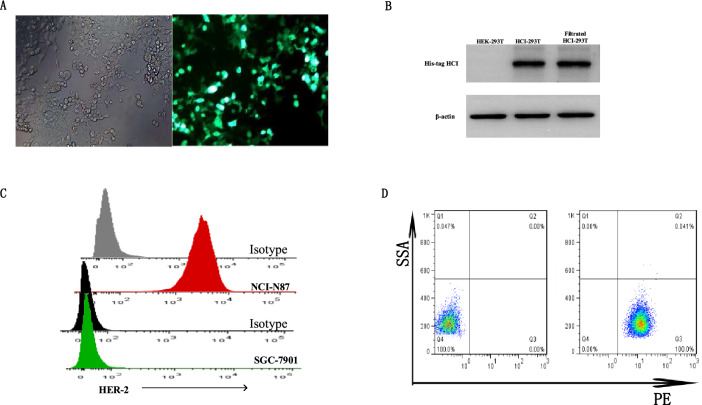
HCI production and HER-2 expression on the gastric tumor cell surface. (**A**) HEK-293 T cells were transfected with HCI fusion protein packing lentivirus containing a GFP cassette. Seventy-two hours after transfection, transfection efficiency was observed under either an optical microscope (left) or fluorescence microscope(right). (**B**) HCI fusion protein expression was determined by Western blot analysis and the blots were cut prior to hybridization with antibodies. (**C**) The expression of HER2 on the tumor cell surface in SGC7901 and NCI-N87 cells was detected by FCM. **(D**) Flow cytometry showed that the HIC fusion protein bound to the HER-2 antigen expressed on the NCI-N87 cell surface (right). NCI-N87 cells treated with the nonHCI fusion protein were used as control groups(left).

To generate the HCI fusion protein targeting HER-2, first, we designed the HCI structure by connecting the HER-2 specific scFv ,CCL19 and IL-7 with linker (Fig. 1a, Supplement Fig. 1). As shown in Fig. 1B, the main mechanism of HCI fusion protein combined with immune cells against HER-2 positive tumor cells is relying on the CCL19 to recruit DC or T cells to migrate to the tumor site and IL-7 can enhance the proliferation and viability of effector cells, which had been approved by the previous studies in 7 × 19 CAR-T cell therapy. Since the lentivirus vector had GFP and puromycin resistance elements, we screened lentivirus-transfected HEK-293 T cells by puromycin and evaluated the screening efficacy by observing the level of green fluorescence. The results showed that the screened HEK-293 T cells had a nearly 100% ratio of green fluorescence (Fig. 2A).

In summary, by transfection with lentivirus and screening with puromycin, we achieved 100% HCI fusion protein expression and sorted HEK-293 T cell strains(HCI-293 T).

### HCI fusion protein with high biological activity can be stably obtained from HCI-293 T cells

HCI fusion protein expression in lentivirus-transduced HCI-293 T cells was assayed by western blot. The HCI fusion protein (63 kDa) was detected in the HCI-293 T lysates but not in the HEK-293 T lysates, indicating successful HCI fusion protein expression in the transduced HCI-293 T cells (Fig. 2B and supplement Fig. 2). ELISA also showed that IL-7 and CCL19 in the enriched HIC fusion protein were biologically active (data not shown). Then, we selected two types of gastric tumor cells,NCI-N87 and SGC7901 for further experiments. HER-2 expression on these two cell lines was detected with FACS, and the results showed 100% and 0%, respectively (Fig. 2C).Then, we performed FACS analysis to evaluate the binding ability of anti-HER-2 scFv to the HER-2 proteins expressed on the surface of NCI-N87 cells. As MIP-3 beta monoclonal antibody can specifically bind to human CCL19, we used the HIC fusion protein as the primary antibody to bind the HER-2 proteins on theNCI-N87 cell surface, and a PE-labeled MIP-3 beta monoclonal antibody was added as a secondary antibody to combine the CCL19 element of the HCI fusion protein. Nearly all of the HCI fusion protein-labeled NCI-N87 cells(100%) were PE-positive, while the non-HCI fusion protein-treated NCI-N87 cells were PE-negative(0%, Fig. 2D). These results indicate that HCI fusion can specifically bind to HER-2 proteins-expressed on the tumor surface and that anti-HER-2 scFv, IL-7 or CCL19 are highly biologically functional.

### HCI fusion protein induced immune cell activation and induced specifically, strong cytotoxicity by PBMCs

**Figure 3:**
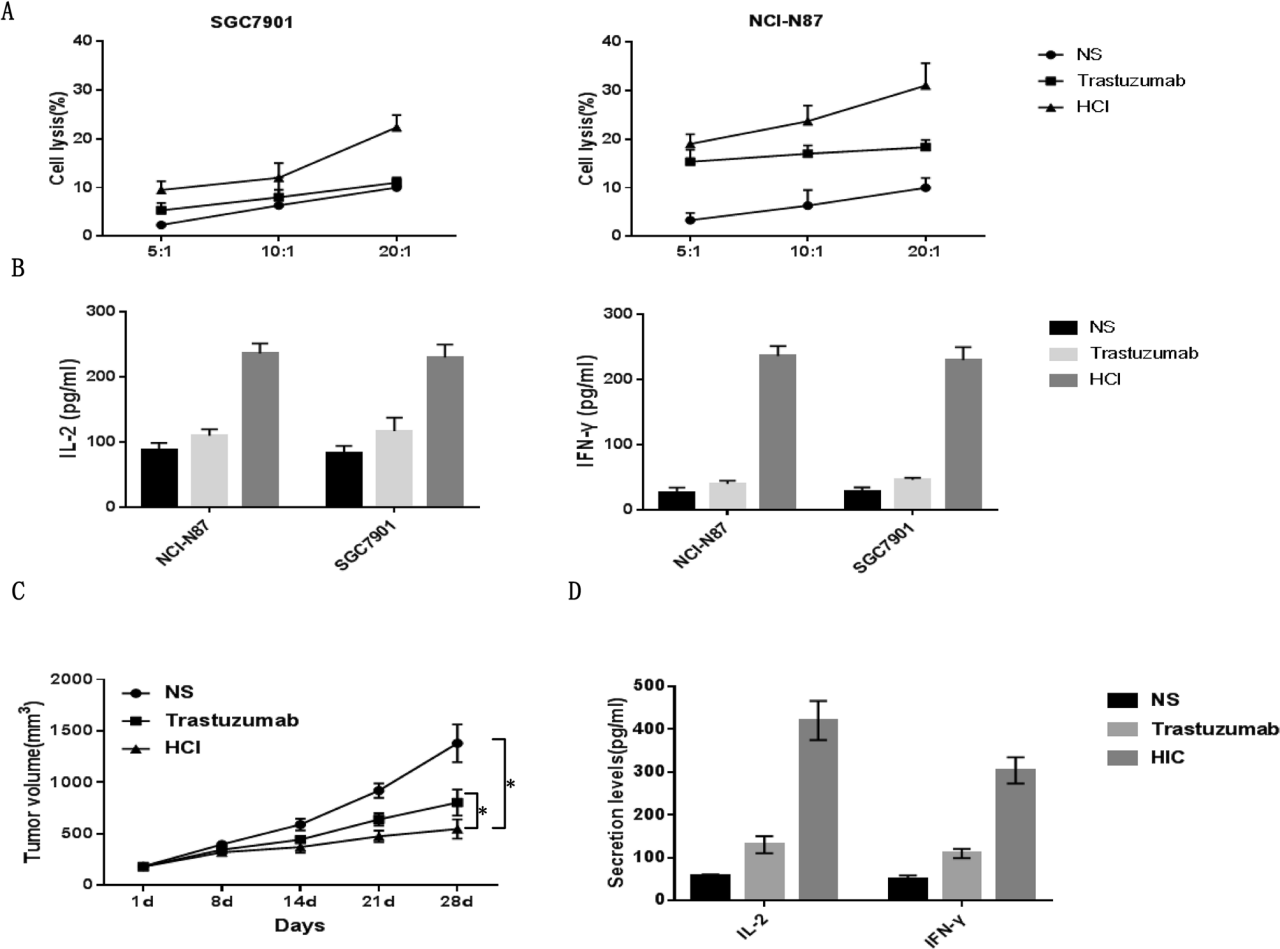
HCI fusion protein-induced immune cells lyse tumor cells both in vitro and in vivo. (**A**) Cytotoxic activity analysis of PBMCs induced by HCI fusion protein. NCI-N87 or SGC7901 cells cocultured with PBMCs at increasing ratios (5:1, 10: 1 and 20:1) in the presence of HCI fusion protein or trastuzumab and CCK-8 assays were used to detect target cell lysates. (**B**) In vitro cytokine secretion (IL-2, IFN-γ) of PBMCs cells analysis. PBMCs co-cultured with target cells at a 10:1 ratio were treated with HCI fusion protein or trastuzumab for 24 h, and ELISA was used for detection. (**C**) After the tumor volume of xenograft mice reached approximately 150 mm3, the mice were treated with standard doses once a week, and the volumes of xenograft mouse tumors were recorded for statistical analysis.(*p < 0.05). (**D**) After the mice were sacrificed, 100 μL of peripheral blood from treated mice was obtained for the in vivo cytokine secretion assay according to the ELISA kit instrument.

To evaluate the in vitro cytotoxic activity induced by theHCI fusion protein, we coincubated MHC-matched PBMCs with HER-2-positive NCI-N87 cells, and HER-2-negative SGC7901 cells were used as a control. The PBMCs of the HIC fusion protein group showed significant cytotoxic activity against NCI-N87 cells, and the cytotoxic efficiency was dose-dependent when compared with that of the trastuzuma or NS group (Fig. 3A right). Impressively, because the HER-2 expression of SGC7901 was negative, we found that the cell lysis rate of the HIC fusion protein-treated groups was higher than that of either the trastuzuma or NS group, indicating that IL-7 can activate T cells in PBMCs (Fig. 3A, left)**.** For the in vitro ELISAs, the IL-2 and IFN-γ cytokine release in the co-incubation medium showed the same tendency as the cytotoxicity assay (Fig. 3B).The above results demonstrated that the HCI fusion protein can induce target cell lysis through antigen-scFv binding and immune cells activated by IL-7.

### HCI fusion protein inhibited HER-2-positive gastric tumor growth in vivo

**Figure 4:**
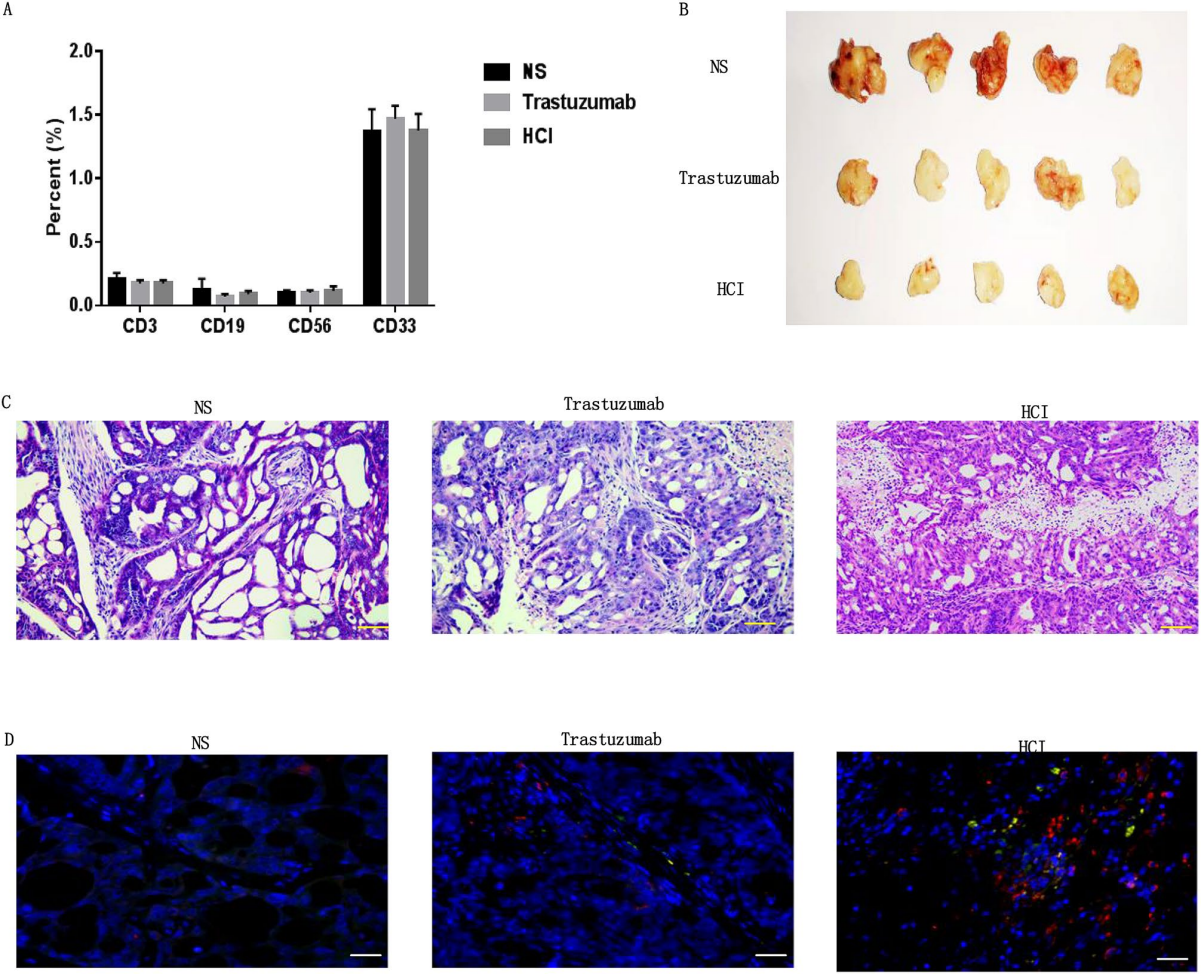
HCI fusion protein mediated autoimmune cell antitumor activity in vivo. (**A**) FACS analysis revealed the presence of memory associated human cells in NOD/SCID mouse peripheral blood 4 weeks after homoimmune reconstitution. (**B**) Tumor tissue images of sacrificed mice with different effector cell treatments are shown. (**C**) After sacrifice, the tumor tissues were obtained and cut into sections for H&E staining. Images of NS (left), trastuzumab (middle) and HCI fusion protein(right)-treated groups of sections are shown, scale bars = 50 μm. (**D**) Immunofluorescence images showing DC (anti-DEC205 staining, green) and T cell infiltration (anti-CD3 staining, red)in the NS(left), trastuzumab (middle) and HCI fusion protein(right)-treated groups, scale bars = 50 μm.

To examine the therapeutic effects of HCI fusion protein in vivo, we applied a humanized immune system mouse model bearing tumor xenografts. The percentage of CD3/CD19/CD56/CD33 in CD45 positve memory associated cells were analysed using FACS (Fig. 4A), suggesting that human immune reestablishment was successful in immune deficient mice. Then the mice were subcutaneously inoculating with 3 × 10^6^ NCI-N87 cells, when the tumor volume reached 150 mm^3^ (d0), each group were treated with either NS, Trastuzumab or HCI fusion protein following standard treatment cycle and drug dose through tail vein injection. After four-times infusion, we observed that the tumor volumes of NS group increased continuously, and 2/5 mice showed ulceration in tumor bearing local skin .Trastuzumab group showed slower tumor growth rate and smaller tumor volumes than that of NS group (*p* < 0.01). In the HCI fusion protein group, tumor volumes were significantly decreased after the third infusion and tumor growth was inhibited consistently, suggesting a strong and long-term tumor-inhibitory effect (Figs. 3C and 4B). The levels of IL-2 and IFN-γ in HCI fusion protein-treated xenograft mice were significantly higher than those in the other groups (Fig. 3D).

### HCI fusion protein-induced immune cell infiltrate into tumor tissues

As the HCI fusion protein can inhibit tumor growth in vivo, we then performed H&E staining and immunofluorescence to determine the antitumor mechanism of the HCI fusion protein (Fig. 4C). Anti-DEC205 and anti-CD3 antibodies were used to reflect the infiltration of DCs and T cells, respectively. The results indicated that the HCI fusion protein-treated tumor tissues had rich infiltration by DCs (green) and T cells(red). For the trastuzumab-treated group, fewer T cells were detected, and there was rare infiltration by DCs (Fig. 4D).These results indicate that the HCI fusion protein can simultaneously recognize the HER-2 antigen of gastric cancer cells and carry effector molecules to tumor tissues, which can induce chemotactic and activated immune molecules to migrate to tumor tissues, break the immune suppression of the tumor microenvironment, and play an antitumor role.

## Discussion

Early gastric cancer shows an excellent prognosis after surgery, while the majority of patients cannot be diagnosed with advanced gastric cancer with distant metastasis. Current treatments for advanced gastric cancer are mainly chemotherapy, and superior methods of cancer treatment are being developed^22^. In addition, several tumor markers, such as HER2, CEA, MUC1, and EpCAM, have been investigated extensively in gastric cancer target preclinical or clinical trials^3^.

Monoclonal antibody therapy is a significant milestone in cancer targeting treatment. The combination of monoclonal antibodies with target antigens is highly specific and can distinguish tumor cells from normal cells. Therefore, compared with chemotherapy drugs, monoclonal antibodies are safe and effective^23^.Trastuzumab, which targets the HER2 protein, is effective in the treatment of breast cancer. Some studies have demonstrated the efficacy and safety of trastuzumab in the treatment of HER2-positive advanced gastric cancer^24^. However, there are a series of obstacles that limit the efficacy of monoclonal antibodies. On the one hand, the main components involved in cellular immunity are T lymphocytes, which are divided into CD8 + cytotoxic T lymphocytes (CTLs), CD4 + helper T cells and regulatory T cells (Tregs). CD8 + cytotoxic T lymphocytes are considered to be the most important antitumor cells. T cells recognize tumor antigens, activate and amplify effector T cells, and secrete cytokines such as IL-2, IL-4, IL-5, IFN-γ and granase to exert cytotoxic killing effects. Among them, IL-2 and IFN-γ had the strongest killing effect, and their secretion was associated with the killing degree^25,26^. The antibody-dependent cytotoxic effect (ADCC) is an important mechanism that induces target cell lysis, and cytotoxic T cells cannot be effectively mediated due to the lack of FcYR I/FcYR III on its surface. On the other hand, the molecular weight of monoclonal antibodies is always larger than that of other drugs^27^, which may makes it difficult to penetrate tumor tissues.

As CAR-T cells that produce IL-7 and CCL19 have shown remarkable antitumor ability in treating various tumors^28^, but the side effects of CAR-T therapy are hard to predict and prevent. Morgan RA reported that a patient with colon cancer metastatic to the lungs and liver was treated with the third generation of HER-2 CAR-T cells and died 5 days after treatment, which is associated with HER-2 antigen expressing on the heart and lung^12^. Thus, we tend to determine whether the recombinant protein can overcome the obstacles of monoclonal antibodies and CAR-T cells. First, we connected the anti-HER-2scFv, CCL19 and IL-7 in series and purified the HCI fusion protein from engineered HEK-293 T cells. Then, we performed a series of assays to verify the specific HER-2 antigen binding ability and biological activities of the HCI fusion protein both in vitro and in a humanized gastric tumor xenograft model. The results demonstrated that the HCI fusion protein can induce specific killing effects by binding to HER-2-positive tumor cells and nonspecific killing effects on HER-2-negative tumor cells by activating T cells in vitro (Fig. 3A). No significant toxicity reactions were observed, and tumor growth was inhibited consistently in the HCI fusion protein-treated groups (Fig. 3D). Immunofluorescence also showed that more DC cells and T cells infiltrated into tumor tissues and caused tumor necrosis (Fig. 4C, D).

These results revealed that the single-chain antibody scFv successfully delivers effector molecules to tumor tissues, which can effectively resist immune suppression by the tumor microenvironment and promote the migration of chemotactic immune cells to tumor tissues and their activation to exert a synergistic antitumor effect. However, single-chain antibodies have only one antigen binding site and have a single function, so there are certain limitations in their use. With the development of genetic engineering technology, there have been single-chain antibody polymers that can specifically bind to multiple antigen determinants, as well as two dual-specific single-chain antibodies that can express different specific antigens. It provides more space for the high efficiency and precision of targeted therapy.

Besides,in our study, we evaluated the HCI activity in the immunodeficiency mice due to the NCI-N87 is a human gastric tumor cell and can not be used to construct the xenograft model in the immunocompetent mice. Although the humanized mice were obtained by infusion the human PBMCs through tail vein injection, these models are difficult to evaluated the immune side-effects and the whole immune response. Future studies are needed to overcome that obstacles by using transgenic mice model for huHER2 as previously described^29,30^.

In conclusion, this study demonstrated that the antitumor activity of HCI was preliminarily studied in vitro and in vivo, and it was confirmed that HCI could mediate the synergistic antitumor effect of the immune system and that the enhanced antitumor ability of the HCI fusion protein produced TME breakthrough effects, providing a new idea for the targeted therapy of different target antigens.

## Supplementary Information

Supplementary Figure 1.

Supplementary Figure 2.

Supplementary Legends.
