# Supplementary material for: Anti-HER2 scFv-CCL19-IL7 recombinant protein inhibited gastric tumor growth in vivo
Source: Sci Rep. 2022 Jun 21;12:10461. doi: 10.1038/s41598-022-14336-1 (PMC9213520; doi:10.1038/s41598-022-14336-1)
Supplement: Supplementary file 3 — Supplementary Legends. [file 41598_2022_14336_MOESM3_ESM.docx]

Supplement Fig 1. Double enzyme electrophoresis of HCI-pcDNA3.1. Line1: Plasmid DNA. Line2: digested with XhoI/HindIII. M:DNA Marker

Supplement Fig 2: Western blots showed the fuller length of blots and the blots were cut prior to hybridization with antibodies.
